# In this month’s Bulletin

**DOI:** 10.2471/BLT.22.000222

**Published:** 2022-02-01

This month’s theme issue focuses on policy and health system responses to the COVID-19 pandemic. In the editorial section, Viroj Tangcharoensathien and Tedros Adhanom Ghebreyesus (90) explain why the timing of the pandemic’s end will be influenced by political choices. Oscar J Mujica et al. (91) emphasise the need to address health inequity in pandemic preparedness and response plans. 

Gary Humphreys (94–95) reports on mobile clinics and their operations, as part of the pandemic response. Uche Amazigo talks to Andréia Azevedo Soares (96–97) about the impact of the pandemic on neglected tropical disease control programmes. 

## Bangladesh

### Are children faring worse?

Sharika Nuzhat et al. (98–107) analyse health and nutritional status data. 

## Haiti, Lesotho, Liberia, Malawi

### Are routine vaccinations being delivered?

### Emilia Connolly et al. (115–126) compare expected to actual levels of coverage.

## India

### How can community health workers contribute?

Sachin S Singh & Lal Bahadur Singh (108–114) document training programmes.

### Are ophthalmological services maintained?

Janani Muralikrishnan et al. (135–143) measure access to eye care.

## Indonesia

### Are mothers and children still receiving health services?

Siti Helmyati et al. (144–154) monitor continuity of service delivery.

## Italy

### How to calibrate the pandemic response?

Flavia Riccardo et al. (161–167) track the effectiveness of rapid risk assessment.

## Philippines

### Are people still getting care for tuberculosis?

Marianne Calnan et al. (127–134) study continuity provided by telephone contact centres.

## Thailand

### How have peritoneal dialysis services adapted?

Talerngsak Kanjanabuch & Krit Pongpirul (155–160) assess consequences for patients.

## United States of America

**Is health equity improving?**

Jazmyn T Moore et al. (171–173) et al detail strategies to reach minority groups. 

## Global

### What have countries done to maintain essential health services?

Nikki Gurley et al. (168–170) capture policy developments.

### How to ensure delivery of health supplies?

Genevie Fernandes et al. (174–176) describe the requirements of resilient supply chains. 

